# Identification of predictive pretreatment biomarkers for neoadjuvant chemotherapy response in Latino invasive breast cancer patients

**DOI:** 10.1186/s10020-025-01338-8

**Published:** 2025-12-09

**Authors:** Hedda Michelle Guevara-Nieto, Rafael Parra-Medina, Carlos A. Orozco, Sandra Diaz-Casas, Jone Garai, Jovanny Zabaleta, Liliana López-Kleine, Alba L. Combita

**Affiliations:** 1 Grupo de Investigación en Biología del Cáncer, Instituto Nacional de Cancerología (INC), Bogotá, 111511 Colombia; 2 Grupo de Investigación Traslacional en Oncología, Instituto Nacional de Cancerología (INC), Bogotá, 111511 Colombia; 3 Doctorado en Oncología, Departamento de Patología, Facultad de Medicina, Universidad Nacional de Colombia, Bogotá, 111321 Colombia; 4 Departmento de Patología, Instituto Nacional de Cancerología (INC), Bogotá, Colombia; 5 Instituto de Investigación, Fundación Universitaria de Ciencias de la Salud (FUCS), Bogotá, 111411 Colombia; 6 Grupo de Seno y Tejidos Blandos, Instituto Nacional de Cancerología (INC), Bogotá, 111511 Colombia; 7 Stanley S. Scott Cancer Center, Louisiana State University Health Science Center (LSUHSC), New Orleans, LA 70112 USA; 8 Department of Interdisciplinary Oncology, Louisiana State University Health Science Center (LSUHSC), New Orleans, LA 70112 USA; 9 Departmento de Estadística, Facultad de Ciencias, Universidad Nacional de Colombia, Bogotá, 111321 Colombia; 10 Departmento de Microbiología, Facultad de Medicina, Universidad Nacional de Colombia, Bogotá, 111321 Colombia

**Keywords:** Invasive breast cancer, Neoadjuvant chemotherapy, Molecular subtypes, Pathological complete response, Tumor microenvironment, Predictive biomarkers, Gene expression profiles

## Abstract

Breast cancer (BC) exhibits significant heterogeneity in incidence and mortality worldwide. Neoadjuvant chemotherapy (NAC) is the standard treatment for locally advanced BC; however, its efficacy varies by subtype. This study examined the gene expression profiles associated with NAC response in Colombian women with invasive BC. Methods: RNA sequencing of pre-treatment tissues from 58 patients (29 responders and 29 non-responders) identified differentially expressed genes (DEGs) for each molecular subtype, and prognostic performance was evaluated using risk scores. Results: Functional enrichment analysis highlighted the immune system pathways in non-responders. Changes in cytokine target activity and immune cell populations were analyzed to understand the role of the tumor microenvironment (TME) in response to treatment. *APOD*,* GPR132*,* FGF10*, and *HBB* emerged as independent predictors of NAC response, with *APOD* showing a protective effect in LuminalB/HER2- patients. These results were corroborated by immunohistochemistry and public databases. Drug sensitivity analysis revealed varied responses to potential therapeutics among the non-responders. Conclusions: This study underscores the need to identify specific gene expression profiles and immune cell population changes to predict NAC responses, paving the way for personalized and effective treatments for the Colombian BC population.

**Supplementary information:**

The online version contains supplementary material available at 10.1186/s10020-025-01338-8.

## Background

Breast cancer (BC) is one of the most common invasive cancers and a leading cause of cancer-related mortality in women (Sung et al. 2020). BC presents high heterogeneity, with incidence and mortality rates that vary among women from different populations worldwide, which affects disease prognosis and response to chemotherapy (Zardavas^2015^). Neoadjuvant chemotherapy (NAC) is the standard treatment for locally advanced BC (Mauri et al. 2005)administered before surgery to reduce tumor burden and provide early response information (Schott and Hayes 2012). This approach extends to stage II/III operable breast cancer to enhance conservative surgical treatment. NAC allows assessment of tumor response to specific chemotherapy, enabling treatment changes in non-responders. Studies have confirmed that tumor reduction after NAC affects disease-free and overall survival. Patients achieving pathological complete response (pCR), indicating no tumor cells in the breast tissue or lymph nodes after surgery, showed better survival than those with residual tumors (Cirier et al. 2017; European^2014^; Cortazar et al. 2014; Wang-Lopez et al. 2015). However, the response to treatment is mainly determined by the pathobiological characteristics of the residual cancer after NAC. Recent studies have indicated significant differences in NAC efficacy among breast cancer subtypes and have suggested that NAC could increase local recurrence despite reduced tumor size (Caparica^2019^; Viale and Fusco 2022). NAC may select resistant clones and promote metastasis through cellular stress and tumor microenvironment (TME) remodeling (Hasim et al. 2018; Perelmuter et al. 2019).

Determining the molecular differences between tumors in order to select the most effective treatment is central to precision oncology. Currently, pCR is a validated surrogate biomarker for survival (Administration 2022), but it is reliable for certain molecular subtypes. This highlights the need to identify predictive biomarkers to accurately identify patients who benefit from chemotherapy and to avoid unnecessary treatment for those likely to be resistant. Therefore, exploring key genes and pathways involved in BC progression and prognosis is crucial. Given the unique genetic and epidemiological traits of Latin American populations, particularly Colombia, it is essential to conduct region-specific studies. Variability in genetic backgrounds, environmental exposure, and access to healthcare can impact tumor molecular profiles and treatment responses. Studying this population will enhance the understanding of tumor biology in this context and help identify relevant predictive biomarkers and tailor precision oncology treatments to improve outcomes. Next-generation sequencing tools have increasingly reported gene expression profiling to identify biomarkers predictive of pCR to NAC in breast cancer. Currently, multigene tests can predict breast cancer recurrence (Derouane 2022); however, evidence for their prognostic value in predicting therapeutic benefits remains limited (Wolff et al. 2008).

The goal of our study was to investigate the genes associated with response to NAC in Colombian women with invasive breast cancer. We hypothesized that elevated expression of these candidate genes would correlate with poor pCR and adverse survival outcomes in patients with BC. By identifying these biomarkers, we aimed to reduce unnecessary treatments in non-responsive cases and to identify specific patient subgroups that may benefit from innovative therapeutic interventions. This study not only addresses the unique genetic and environmental factors of the Colombian population but also seeks to enhance the precision and efficacy of breast cancer treatment within this demographic.

## Methods

### Breast cancer tissue collection

This IRB-approved study (Protocol C1901300403) included 444 women with locally advanced mammary adenocarcinoma (stage IIB to IIIC) diagnosed at the National Cancer Institute of Colombia between September 2013 and March 2021. Participants who provided informed consent were newly diagnosed with non-metastatic ductal breast cancer and treated with NAC followed by surgery. Exclusion criteria were pregnancy, breastfeeding, history of carcinoma, previous chemotherapy or radiotherapy, and nonstandard treatments. Clinical and sociodemographic data were obtained from the institute’s Functional Breast Cancer Unit and electronic health records.

###  Breast cancer sample collection

To explore gene expression profiles associated with NAC non-responsiveness, a discovery set of 58 pre-treatment formalin-Fixed Paraffin-Embedded (FFPE) biopsy samples was categorized into 29 responders and 29 non-responders. A pathologist evaluated each sample to identify tumor tissue and assess response in post-NAC specimens. Responders were defined as those achieving pathological complete response (pCR), characterized by the absence of invasive tumor cells in both the breast and lymph nodes (ypT0/is ypN0). Non-responders include patients with any residual invasive disease in the breast and/or lymph nodes. To enhance RNA quality, FFPE samples with > 90% tumor sections were punched; otherwise, manual microdissection was performed.

### RNA extraction

FFPE samples were deparaffinized using xylene and ethanol, followed by total RNA extraction using the Qiagen All Prep DNA/RNA FFPE Kit (Germany) and MagMAX^®^ FFPE RNA Ultra Kit (USA), following the manufacturer’s protocol. Extracted RNA was frozen at −80 °C, and its quantity and quality were assessed using a Qubit3.0 and an Agilent2100 Bioanalyzer. The DV200 value, representing the RNA percentage of at least 200 nucleotides long, was calculated using Bioanalyzer software.

### Library preparation and sequencing

Genomic libraries were prepared using the Illumina TruSeq RNA Exome Library prep kit with modifications for highly degradable FFPE material (Wen 2017): 300ng RNA input, DV200 > 30% samples, nine PCR cycles, and 16-hour hybridization. Quantification was performed using Qubit and Agilent Bioanalyzer 2100. Standardized 10 nM libraries were pooled, denatured, mixed with PhiX control, clustered, and sequenced in a NextSeq500 High-Output flow cell at 2 × 75 bp, yielding > 90% sequencing quality and an average of 30,105,535 reads per sample.

### RNA sequencing data processing, gene expression analysis of differentially expressed genes (DEGs)

Partek Flow software (Partek, St. Louis, MO, USA) was used for genome alignment and quality control. Reads were trimmed, contaminants removed (Bowtie 2 v2.2.5), and aligned to the human genome (hg38) using STAR 2.7.3a. Quantification was performed using RefSeq Transcripts 96 and genes with at least five reads in 80% of the samples were retained. DEseq2 was used for differential expression analysis after normalization (VST), applying Benjamini and Hochberg correction (FDR = 0.05) to identify DEGs. Gene expression was compared across intrinsic subtypes: Luminal A, LuminalB/HER2-, LuminalB/HER2+, HER2-enriched, and Triple-negative Breast Cancer (TNBC), in response to neoadjuvant chemotherapy. Library preparation, sequencing, and analysis were performed using the Translational Genomics Core (LSUHSC).

### Intersection of DEGs with oncogene lists

The “InteractiVenn” webtool (Heberle 2015) identified intersecting DEGs with OncoKB database (Chakravarty 2017; Suehnholz et al. 2024), which lists curated oncogenes and tumor suppressor genes associated with various cancers. Functional insights into these genes are based on experimental evidence. EnrichR tool (Xie et al. 2021) analyzed gene ontology (GO) term enrichment for intersecting DEGs, covering biological processes and pathways involving these genes. A Benjamini-Hochberg FDR < 0.05 was the cutoff criterion.

### Intersection with commercial genomic panel tests

To assess the predictive and prognostic value of chemotherapy in our cohort, we compared our DEG lists with genes from genomic tests such as MammaPrint, EndoPredict, Oncotype DX, and PAM50. Gene lists from Derouane et al. (Derouane 2022) were analyzed to identify shared molecular signatures, potentially enhancing our understanding of tumor behavior and therapeutic outcomes.

### Evaluation of gene expression profiles associated with prognosis

The Tumor Immune Estimation Resource (TIMER) web server tool (Li et al. 2017) was used to evaluate DEGs and analyze their correlation with survival in The Cancer Genome Atlas Breast Invasive Carcinoma (TCGA-BRCA) patients. Using all DEGs identified, we performed a Cox proportional hazards regression analysis to evaluate the association between gene expression levels and overall survival, utilizing the TIMER tool. Genes were filtered based on statistical significance, including only those with an adjusted p-value < 0.05. This approach ensured the selection of genes whose overexpression was associated with an increased risk of poor survival outcomes. We used the TCGA-BRCA survival data to calculate the risk scores. For each patient, we multiplied the regression coefficient of each gene (from TCGA) by its respective normalized expression counts from our study cohort to quantify the strength of the association between gene expression and survival risk. The products of these calculations were used to generate an overall risk score for each patient, reflecting the cumulative impact of the gene expressions on patient survival risk. The median risk score was calculated across the cohort, and patients were subsequently classified into high-risk and low-risk groups based on whether their risk score was above or below the median. Risk score was calculated as follows:

$$Risk\;Score\;=\;\sum\limits_{i=1}^{\left\{n\right\}}\;\left(\beta_i\;\times\;Expression_i\right)$$

Kaplan-Meier curves were estimated using Survminer package (Kassambara 2022), responders and non-responders were divided into high- and low-expression groups based on median gene expression levels. Overall survival (OS) was measured from the time of study entry to death. Logistic regression models were used to evaluate the predictive performance of baseline values and percentage changes for each parameter, with binary pCR outcomes as the dependent variable. The area under the receiver operating characteristic curve (AUC-ROC) was used to assess the predictive performance. A combined gene risk score was calculated by weighting each gene (0.5 assumption) and summing the products of these weights and gene values (Yan et al. 2018). Univariate and multivariate logistic regression models incorporated clinical, demographic, and genetic features, with statistical significance set at*p* < 0.05. The ‘caret’ and ‘pROC’ packages were used for logistic regression and ROC analysis, respectively.

### Incorporating bootstrap and jackknife approaches for small sample size

To address the small sample size and enhance reproducibility, sensitivity analyses were performed using bootstrap resampling and jackknife methods. Specifically, 20 bootstrap iterations were carried out under two sampling schemes: (Sung et al. 2020) 6 nonresponder and 6 responder samples, and (Zardavas 2015).11 nonresponder and 6 responder samples, both randomly sampled with replacement. These scenarios allowed for the evaluation of consistency across different sample compositions. For each iteration, differential gene expression analysis was performed to identify the top 20 shared differentially expressed genes across the bootstrap schemes. To assess the variability and consistency of gene expression across assays, several statistical measures were calculated for each gene. The mean fold change was computed by averaging the fold-change values across all iterations, ignoring missing values. The standard deviation of fold changes was calculated to quantify the variability in gene expression. The coefficient of variation was then derived by dividing the standard deviation by the absolute value of the mean fold change, providing a relative measure of variability. Additionally, the number of valid data points was calculated by counting the non-missing fold-change values for each gene, ensuring that the analysis accounted for missing data across assays. Additionally, the jackknife method was applied to assess the bias and variance of the estimates, thus improving the generalizability of the results (Efron and Gong 1983; Zhang^2010^; Taylor 1991; Tukey 1958). The combination of these methods enabled the assessment of the stability of gene expression across assays. To evaluate the consistency of fold-changes, boxplots and heatmaps were generated to identify genes with consistent expression patterns, thereby minimizing the potential impact of small sample size.

### External gene expression confirmation

Microarray expression profiles of several datasets were downloaded from the Gene Expression Omnibus (GEO) database. The keywords “neoadjuvant chemotherapy AND breast cancer AND non-responder” were used in the search, and the results were limited to *Homo sapiens*. The inclusion criteria for the search were as follows: (Sung et al. 2020) at least 30 gene expression profiles; (Zardavas^2015^) data obtained for the same cancer type using the same experimental platform; (Mauri et al. 2005) every profile linked with the patient’s clinical history; (Schott and Hayes 2012) all cancers treated with at least one common drug or chemotherapy regimen; and (Cirier et al. 2017) available treatment outcomes enabling the classification of every case as either responders or non-responders. *APOD* expression between non-responders and responders was identified using GEO2R web tool. *P* < 0.05 adjusted to Benjamini & Hochberg (false discovery rate) was chosen as the cut-off criterion.

### Immunohistochemical validation

Tumor sections (4 μm) were prepared, antigen retrieved, and treated with hydrogen peroxide. Anti-APOD (ab 256496; Abcam) primary antibody was applied, followed by biotinylated secondary antibodies, diaminobenzidine, and hematoxylin counterstaining. Controls included omitting primary antibody (negative), benign breast (APOD-positive) sections. A blinded pathologist assessed the APOD-positive staining intensity (weak, moderate, or strong) and the percentage of malignant cells.

### Functional enrichment analysis of DEGs

Pathway enrichment analysis using the Kyoto Encyclopedia of Genes and Genomes (KEGG)(Kanehisa et al. 2010), Gene Ontology (GO) (Ashburner et al. 2000), and Disease Ontology (DO)(Osborne et al. 2009) was conducted using the clusterProfiler R package (Yu et al. 2012) with DEGs from RNA-Seq data to identify pathways in nonresponder patients. False discovery rate (FDR) < 0.05 was chosen as the cutoff criterion.

### Cytokine target activity

CytoSig online tool (Jiang et al. 2021) predicted cytokine target activity using normalized expression data, yielding a Z-score activity. This platform offers a database for studying cellular responses to signaling molecules.

### Estimation of relative cell type abundance

Cell type abundance in the tumor microenvironment (TME) was estimated using xCell (Aran 2017) (64 immune and stromal cells) and CIBERSORTx (Newman et al. 2019) (22 immune cells) on normalized RNA-seq data. All sequenced samples were processed using xCell and CIBERSORTx to calculate per-sample and between-sample stroma and immune scores for non-responders and responders using the non-parametric Mann-Whitney U test with FDR correction for multiple comparisons.

### Drug sensitivity analysis (pRRophetic analysis)

To predict and suggest chemotherapy and targeted agents efficacy in BC treatment, we used the “pRRophetic” R package (Geeleher 2014). This tool estimates the half-maximal inhibitory concentration (IC50) of drugs using statistical models based on drug sensitivity and gene expression data. The Wilcoxon signed-rank test assessed IC50 differences between responders and non-responders.

## Results

### Clinicopathologic characteristics of locally advanced breast cancer patients

Of the 444 patients with locally advanced tumors (Supplementary Fig. 1), 176 were excluded due to incomplete NAC treatment, relocation, or insufficient tissue in their FFPE blocks for RNA extraction. RNA was extracted from FFPE material of 268 patients who completed the NAC regimen; however, 62 samples had insufficient RNA concentrations for sequencing. Thus, 206 patients were included in the study, of whom 140 (67.9%) did not achieve pCR after NAC. Supplementary Table 1 details the clinicopathological characteristics of the discovery (sequencing, *n* = 58) and validation (*n* = 148) cohorts, and shows no significant differences between them. The median age of patients in both groups was 53 years. Despite BMI differences in the validation cohort, most patients were overweight or obese. The majority were postmenopausal and classified as grade II or III, with clinical stage IIIB being the most common. The distribution of breast cancer subtypes between response groups was not statistically significant. Most patients did not have metastatic nodules, and the percentage of live women among responders was not statistically significant.

### Transcriptome profiles of breast cancer patients differ between non-responders and responders

**Fig. 1:**
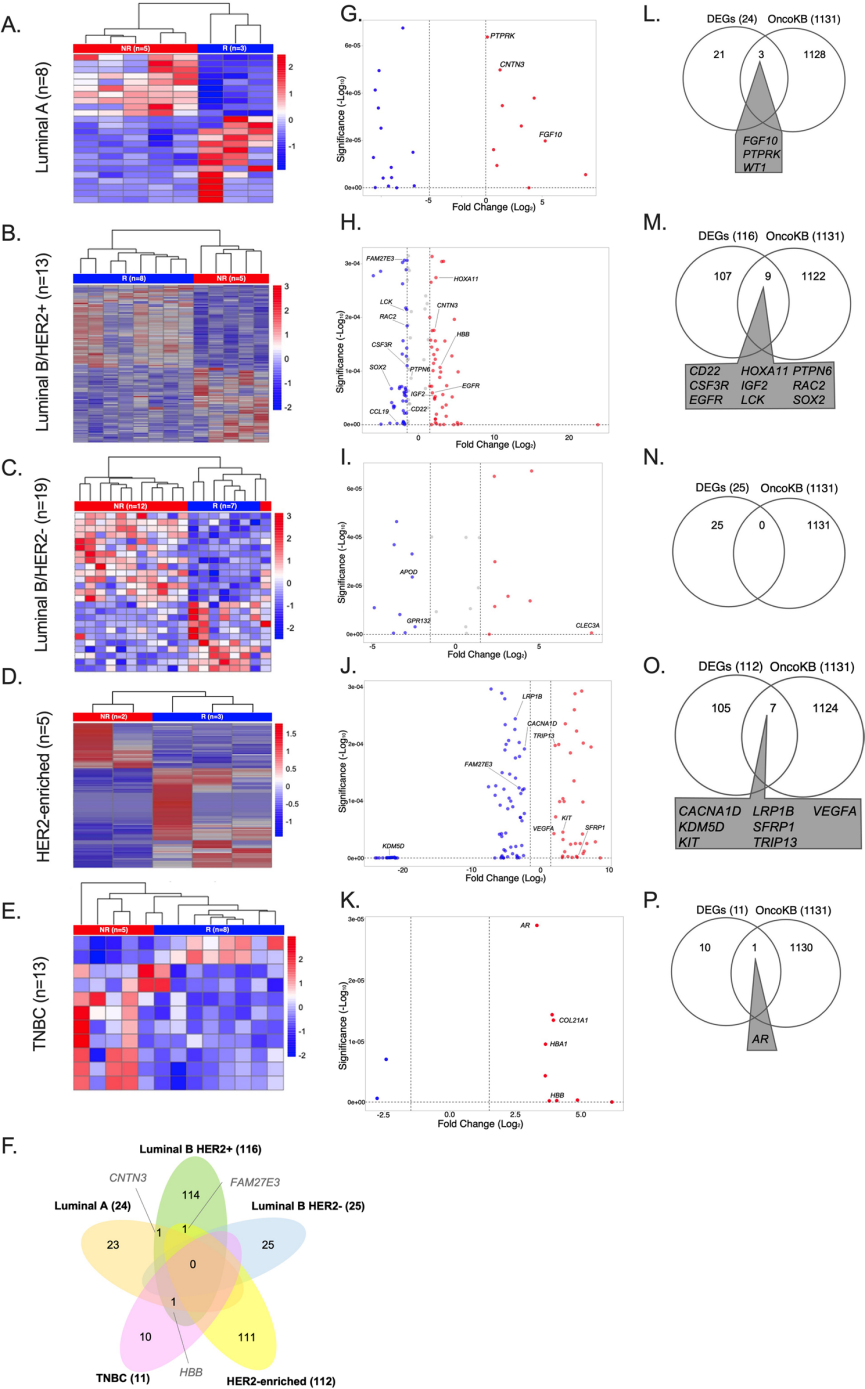
Differential expression patterns and analysis of predictive NAC response DEGs across subtypes. Expression heatmap of prognostic genes between baseline samples from responders (light green bar) and non-responders (light red bar) (**A-E**) among different molecular subgroups. Venn diagram depicting all shared DEGs among molecular subtypes (**F**). Volcano plot showing selected DEGs between Responders and Non-responders among molecular subtypes (**G-K**). Venn diagrams depicting shared DEGs among molecular subgroups and oncogene lists (OncoKB database) (**L-P**). The heatmap shows the supervised cluster of samples using Euclidean correlation distances of differentially expressed genes, considering a fold change > 2 or <−2 and FDR < 0.05. The color scale from red to blue represents the gradient from high to low expression. Columns represent samples or patients and rows represent individual transcripts (differentially expressed genes). The gene expression values (TPM) shown in the heatmap are z-scores normalized for each gene. The volcano plot shows that blue dots represent decreased expression, red dots represent increased expression, and grey dots represent unchanged gene expression. Venn diagrams show all the DEGs in parentheses. Luminal A (**A, G, L**), LuminalB/HER2+ (**B, H, M**), LuminalB/HER2- (**C, I, N**), HER2-enriched (**D, J, O**), and TNBC (**E, K, P**). LuminalB/HER2- did not have shared genes with oncogene lists and TNBC only one gene

Transcriptome profiling via RNA-Seq identified key DEGs for predicting NAC response. Unsupervised clustering analysis showed distinct gene expression patterns between non-responders and responders across molecular subtypes (Fig. 1). Hierarchical clustering effectively segregated baseline samples of responders and non-responders in Luminal A (Fig. 1A), LuminalB/HER2+ (Fig. 1B), LuminalB/HER2- (Fig. 1C), HER2-enriched (Fig. 1D), and TNBC (Fig. 1E). DEGs for each subtype are listed in Supplementary Table 2.

Some DEGs were shared among breast cancer subtypes (Fig. 1F). *CNTN3* was common to Luminal A (Fig. 1G) and LuminalB/HER2+ (Fig. 1H), *FAM27E3* was shared by LuminalB/HER2+(Fig. 1H) and HER2-enriched (Fig. 1J), and *HBB* was identified in both LuminalB/HER2 + and TNBC samples (Fig. 1K). *CNTN3* and *HBB* were overexpressed, whereas FAM27E3 was downregulated. No DEGs were common to LuminalB/HER2- or all subtypes. However, some DEGs (*APOD*,* AR*,* CACNA1D*,* CDC42BPA*,* CD22*,* CLEC3A*,* CSF3R*,* EGFR*,* FGF10*,* GPR132*,* HOXA11*,* IGF2*,* IGFBP5*,* KDM5D*,* KIT*,* LCK*,* LRP1B*,* PTPN6*,* PTPRK*,* RAC2*,* SFRP1*,* SOX2*,* TRIP13*,* VEGFA*,* WT1*) were specific to each subtype.

### Intersection of DEGs with oncogene lists

To determine whether DEGs identified across molecular subtypes were previously reported oncogenes, we used the OncoKB database for intersection and functional enrichment analyses. Three intersecting DEGs were identified as oncogenes for Luminal A (*FGF10*,* PTPRK*,* and WT1)*, and were involved in keratinocyte proliferation and DNA metabolic process regulation (Fig. 1L, Supplementary Fig. 2 A). LuminalB/HER2 + had *CD22*,* CSF3R*,* EGFR*,* HOXA11*,* IGF2*,* LCK*,* PTPN6*,* RAC2*,* and SOX2* linked to MAPK cascade regulation, intracellular signal transition, and cell differentiation pathways (Fig. 1M, Supplementary Fig. 2B). No intersecting DEGs were found in LuminalB/HER2− (Fig. 1N). *CACNA1D*,* KDM5D*,* KIT*,* LRP1B*,* SFRP1*,* TRIP13*,* and VEGFA* were observed in HER2-enriched and involved in focal adhesion, androgen receptor signaling, and cell chemotaxis pathways (Fig. 1O, Supplementary Fig. 2 C). *AR* was found only in TNBC, and no associated pathways or interactions were observed (Fig. 1P).

### Intersection with commercial genomic panel tests

Intersection analysis was performed to determine whether DEGs were part of the genomic tests for BC recurrence risk. An overlap was found only between genes in the MammaPrint and PAM50 tests, and those identified as differentially expressed in our study for LuminalB/HER2 + and HER2-enriched subtypes (Supplementary Table 3). *CDC42BPA* (Mammaprint) and *EGFR* (PAM50) were common in LuminalB/HER2+. For HER2-enriched tumors, Mammaprint *IGFBP5* and PAM50 *SFRP1* matched our DEG list.

### Evaluation of gene expression profiles associated with prognosis

**Fig. 2:**
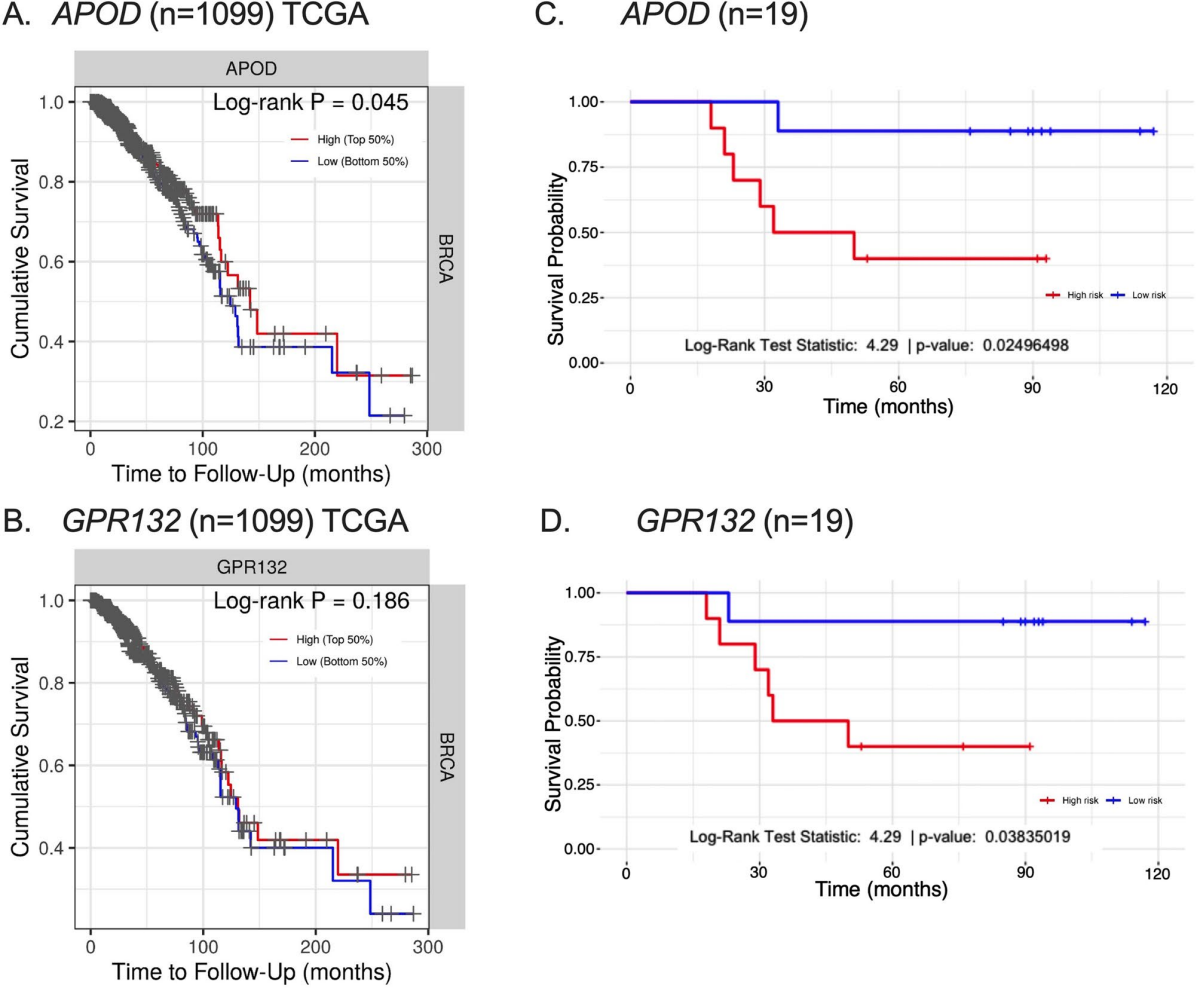
Survival analysis based on the risk prognostic model. KM survival curve for patients in the low- and high-risk groups in LuminalB/HER2- stratified by the gene median value of expression. **A** *APOD* from TCGA cohort, **B ***APOD* from our discovery cohort, **C** *GPR132* from TCGA cohort, and **D** *GPR132* from our discovery cohort. Statistically significant differences were observed (*p* < 0.05)

To assess the prognostic performance of all DEGs across molecular subtypes (Supplementary Table 2), we calculated the risk score using RNA-seq data from the TIMER database. DEGs’ expression levels were split into low and high categories using the median risk score as a cutoff. Survival probability was analyzed by classifying patients according to their gene expression levels. No significant difference was found for Luminal A, LuminalB/HER2+, HER2-enriched, and TNBC subtypes; however, a trend of worse survival in the high-risk group was observed (Supplementary Fig. 3 and 4). In the LuminalB/HER2- subtype, *APOD* and *CLEC3A* expression levels showed that low levels correlated with better prognosis (*p* < 0.05, Fig. 2). Patients with *APOD* expression above the median were classified as low-risk because of its association with a better response.

To assess the prognostic value of DEGs, we conducted univariate analyses alongside clinical parameters in our cohort. Clinical stage IIIC, as well as *FGF10*, and *HBB* expression were significantly associated with a higher likelihood of non-response (Supplementary Table 4). In contrast, *GPR132* and *SOX2* were associated with a reduced risk of nonresponse. *GPR132* showed a protective effect in Luminal B/HER2- patients, whereas *CCL19* and *CSF3R* showed protective effects in Luminal B/HER2 + patients.

**Fig. 3:**
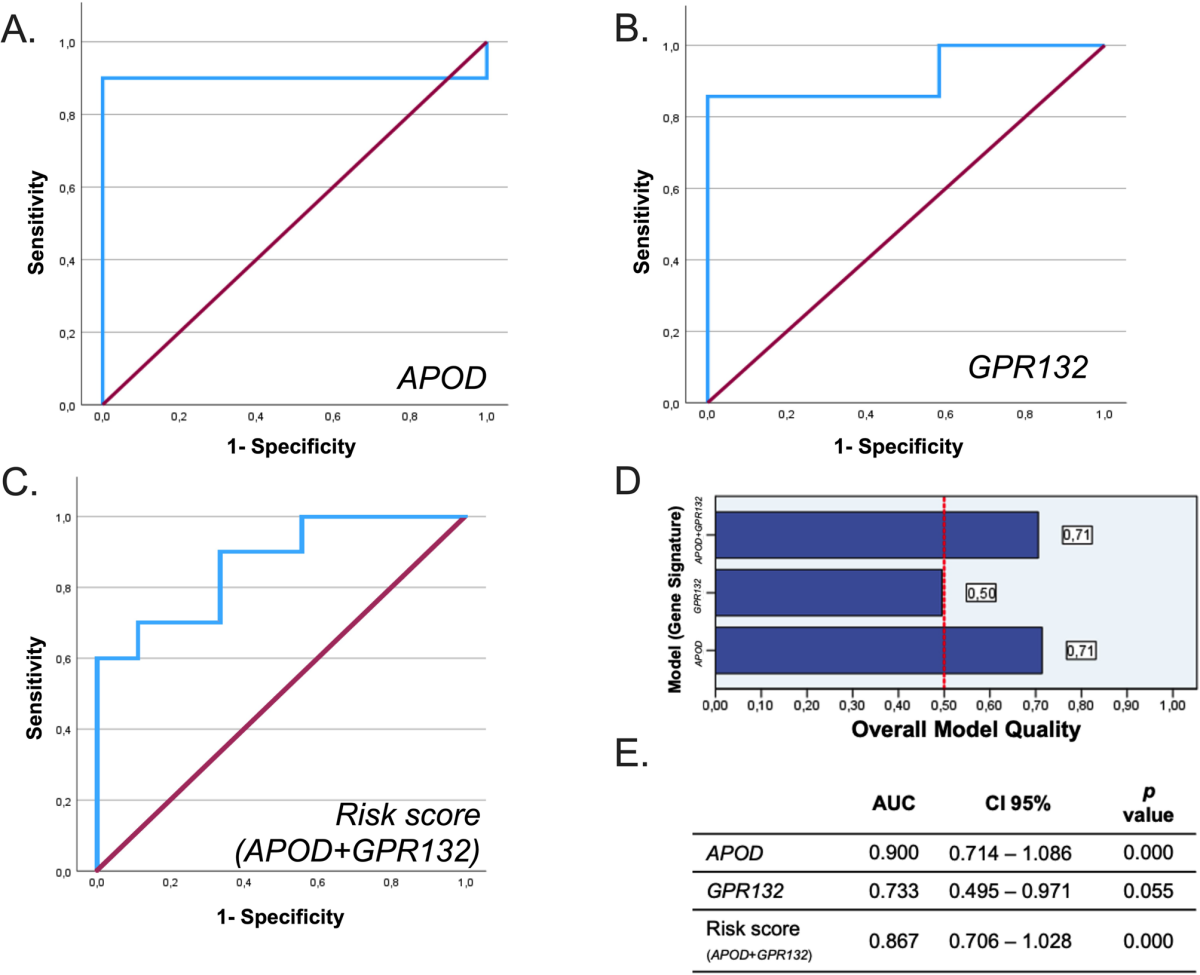
Receiver operating characteristic (ROC) curve analysis for predicting pCR for APOD, GPR132 and combined APOD + GPR132. ROC curves of gene signatures for predicting pCR for (**A**) *APOD*,(**B**) *GPR132*, and (**C**) combined *APOD* + *GPR132*. **D** Overall model quality of the gene signatures. **E** AUC and p-values for gene signatures

We compared our results with those of the entire TCGA cohort for *APOD* (Fig. 2A) and *GPR132* (Fig.  2C). *APOD* (coef:−0.085, HR: 0.918, 95% CI:0.858–0.983, log-rank test *p* = 0.045), *GPR132* (coef: −0.214, HR: 0.807, 95% CI:0.664–0.981, log-rank test *p* = 0.186). Cox proportional hazard model demonstrated a hazard ratio of 0.9 (*p* < 0.05) for *APOD*, suggesting that each unit increase in its expression is linked to a 10% reduction in risk of non-response, indicating a potential protective role in breast cancer. As shown in Fig.  2, a low-risk score correlated with improved prognosis (*p* < 0.05). Specifically for TCGA BRCA-luminal cohort, the analysis for APOD and GPR132 did not reveal statistically significant results (Supplementary Fig. 5). The model demonstrated strong predictive performance, with AUC values of 0.90, 0.73 and 0.87 for *APOD*,* GPR132*, and the combined score, respectively (Fig. 3). Using a cut-off of 0.5, which maximized the highest Youden’s J statistic (0.69), *APOD* showed a sensitivity of 0.83 and specificity of 0.86. For *GPR132*, a cut-off of −4.143 yield a sensitivity of 0.833 and a specificity of 0.857. Although both *APOD* and *GPR132* were identified as significant genes in our initial cohort, survival analysis using the TCGA dataset revealed that only *APOD* demonstrated a statistically significant association with overall survival. *GPR132* did not reach statistical significance in the TCGA cohort (log-rank *p* = 0.186) and did not enhance the prognostic power when combined with *APOD* (Fig. 3E). Therefore, while *GPR132* showed potential within our cohort, subsequent analyses and model construction focused primarily on *APOD*, which exhibited stronger prognostic specificity and sensitivity across datasets.

### Bootstrap analysis of gene expression variability in non-responders and responders

To assess the reproducibility of gene expression in response to chemotherapy, a bootstrap resampling approach was employed using two different sampling schemes: (Sung et al. 2020) 6 non-responders and 6 responders (*n*= 12), and (Zardavas 2015) 11 non-responders and 6 responders (*n* = 17). The bar plots in Supplementary Fig. 6 (Panels A, D) illustrate the fold-change variability (standard deviation, SD) across 20 bootstrap iterations for each gene. *CLEC3A* displayed the highest fold-change variability in both sampling schemes, particularly in assays with non-responder samples, indicating substantial variability in its expression. The box plots (Panels B, E) show that *CLEC3A* exhibited significant fluctuations in fold-change across assays, with large differences observed between responders and non-responders, suggesting that its expression is strongly influenced by sample composition. *APOD*, in contrast, demonstrated more consistent expression across assays, as indicated by lower variability in Panel D (11 non-responders and 6 responders) and moderate variability in Panel A (6 non-responders and 6 responders). The box plots (Panels B and E) reveal moderate but stable fold-change distributions for *APOD*, with fewer outliers compared to *CLEC3A*. Heatmaps (Panels C, F) show consistent differential expression patterns for both genes, but *CLEC3A* shows stronger upregulation in specific assays, while *APOD* exhibits a more moderate and consistent pattern of expression across all assays, with minor fluctuations between non-responders and responders. These analyses highlight that *CLEC3A* is a more variable marker, while *APOD* exhibits greater stability, confirming their differential roles in the context of chemotherapy response.

### GEO datasets analysis

Several Gene Expression Omnibus (GEO) datasets were selected to confirm *APOD* expression. There was a significant differential expression of *APOD* in GSE20194 and GSE25066 datasets (Supplementary Table 5).

### Immunohistochemical validation

**Fig. 4:**
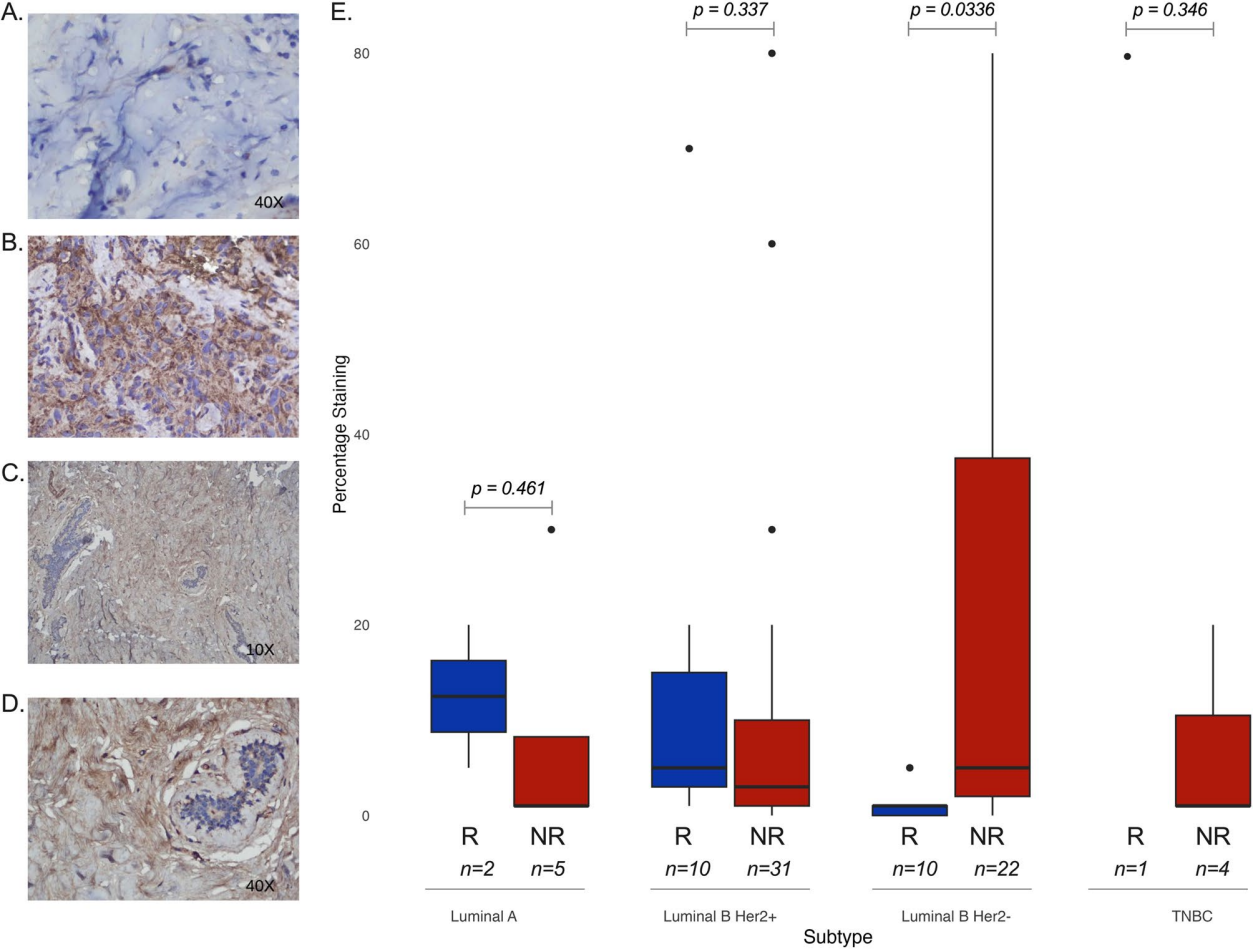
The expression of APOD (in responders and non-responders was determined using immunohistochemistry. APOD Protein expression in non-responders in breast cancer (x40X) (**A**), responders (x40X), positive controls for APOD (10X, **C**), (40X, **D**). Boxplot of percentage staining by response within subtypes (**I**)

With a cut-off point of 1% for APOD positivity, APOD expression in malignant cells was observed in 59 of 85 (69.4%) samples analyzed. A higher expression was observed in non-responders (78%, 46/59) than in responders (22%, 13/59). When analyzed by subtype, only LuminalB/HER2- (*n* = 32) showed significant differences between responders and non-responders (*p* = 0.03) (Fig. 4A-E).

### Functional analysis of RNAs in non-responder patients to NAC

**Fig. 5:**
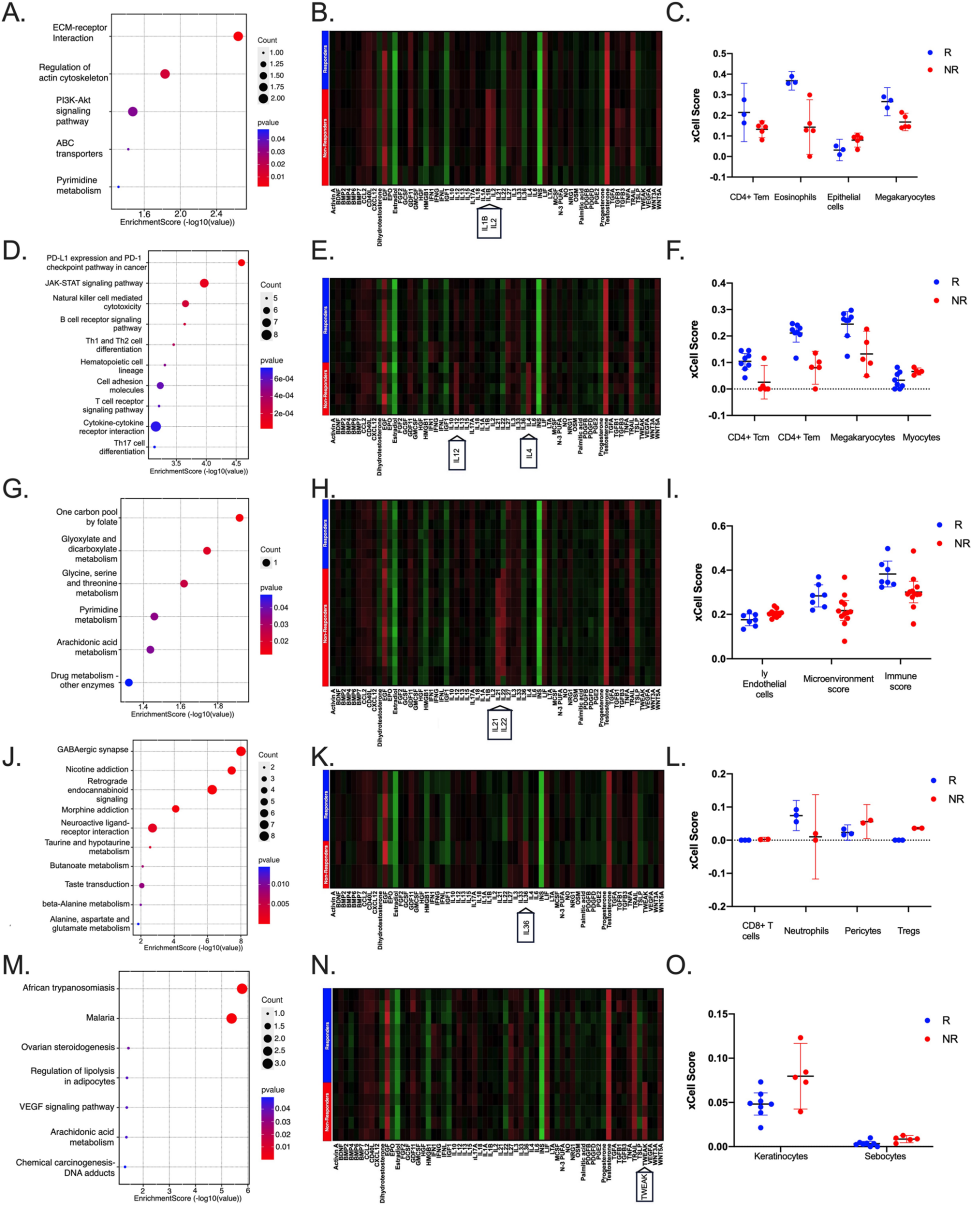
Enriched pathways, cytokines, and tumor microenvironment profiles between non-responders and responders among BC molecular subtypes. Functional analysis (significantly enriched pathways) of differentially expressed genes in baseline samples between non-responders and responders among different BC molecular subtypes (**A**, **D**, **G**, **J**, and **M**). Cytosig heatmaps of cytokine target activities predicted across all molecular subtypes between Responders and Non-responders (**B**, **E**, **H**, **K**, and **N**). Significant cellular heterogeneity landscape of tissue expression profiles among different molecular subgroups using the xCell (**C**, **F**, **I**, **L**, **O**). Luminal A (**A**-**C**), LuminalB/HER2+ (**D**-**F**), LuminalB/HER2- (**G**-**I**), HER2-enriched (**J**-**L**) and TNBC (**M**-**O**). *P* < 0.05; as determined by non-parametric U-Mann Whitney between responders (blue) and non-responders (red). Dotplot rows in A, D, G, J, and M show the top10 most enriched Kyoto Encyclopedia of Genes and Genomes (KEGG) pathways. The more significant adjusted p-value is indicated by the intensity of the red color, and the enrichment level of pathways is indicated by the enrichment score and size of the dot (gene count), as indicated. *P* < 0.05, as determined by the non-parametric Mann-Whitney U test

To understand the underlying functional mechanisms involved in treatment response, we conducted Gene Ontology enrichment analysis using the DEGs list from the two groups of patients. The top 10 functionally enriched biological processes obtained from clusterProfiler analysis under Gene Ontology terms are indicated in the dot plot of the DEGs obtained from the different comparison group analyses (Fig. 5A, D, G, J, M), as well as DEGs for each subtype (Supplementary Table 6).

### Cytokine target activity

TME cytokines, chemokines, and growth factors shape immune cell fate, directing key processes, such as proliferation, growth, and phenotypic changes (Hinshaw and Shevde 2019). CytoSig was used to assess the relationship between gene expression and specific cytokines that could lead to non-response to treatment. Elevated levels of *IL-1B* and *IL-2* were observed in Luminal A non-responders (Fig. 5B), while *IL-12* and *IL-4* were more expressed in LuminalB/HER2 + non-responders (Fig. 5E). Similarly, *IL-21* and *IL-22* exhibited higher expression levels in LuminalB/HER2- non-responders (Fig. 5H). In the HER2-enriched subtype, *IL-36* expression was elevated in non-responders (Fig. 5K), and *TWEAK* was highly expressed in TNBC non-responders (Fig. 5N).

### Estimation of relative cell type abundance

As several enriched pathways were associated with the immune system and TME, enrichment and deconvolution analyses using xCell and Cybersort algorithms, respectively, were performed to predict and characterize changes in immune cell populations related to treatment response. xCell uses gene signatures from 64 cell types, including adaptive and innate immune cells, hematopoietic progenitors, epithelial cells, and extracellular matrix cells, derived from thousands of expression profiles (Aran 2017). As shown in Fig. 5C for Luminal A, significant differences were observed in CD4 + effector memory T cells (CD4 + Tem), eosinophils, epithelial cells, and megakaryocytes on xCell, whereas only differences were observed in CD4 naïve T cells for CIBERSORTx (Supplementary Fig. 7 A). For LuminalB/HER2 + cells, xCell showed significant changes in CD4 + central memory T cells (CD4 + Tcm), CD4 + effector memory T cells (CD4 + Tem), megakaryocytes, and myocytes (Fig. 5F), whereas CIBERSORTx showed differences in Macrophages M0 (Supplementary Fig. 7B). LuminalB/HER2- only showed differences using xCell in lymphatic endothelial cells (ly endothelial cells), microenvironment, and immune scores (Fig. 5I). In HER2-enriched cells, differences were observed in CD8 + T cells, neutrophils, Pericytes and Regulatory T-cells (Tregs) on xCell (Fig.  5L) and Macrophages M0, resting mast cells, and NK cells using CIBERSORTx (Supplementary Fig. 7 C). TNBC samples showed differences in keratinocytes, sebocytes for xCell (Fig. 5O), and T cells CD4 memory activated, monocytes, and dendritic cells (Supplementary Fig. 7D). The remaining immune and stromal cell populations showed no significant difference (Supplementary Table 7).

### Drug sensitivity analysis (prrophetic analysis)

**Fig. 6:**
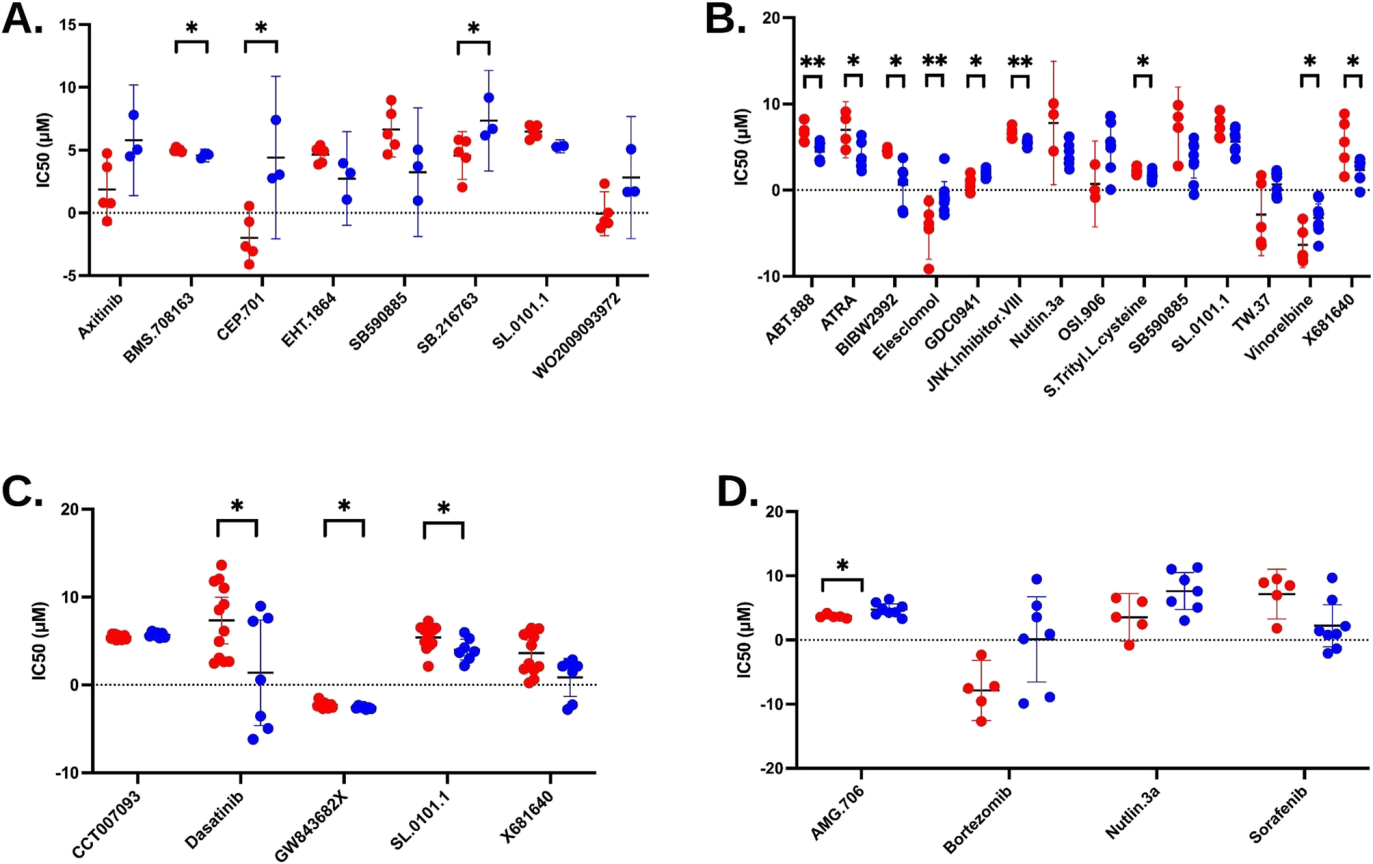
Prediction of clinical chemotherapeutic response from pre-treatment tumor gene expression levels. Sensitivity to chemotherapeutic drugs in (**A**) Luminal A (**B**) LuminalB/HER2+, (**C**), LuminalB/HER2-, (**D**) TNBC from non-responders (red) and responders (blue) breast cancer patients. The half-maximal inhibitory concentration (IC50) of the commonly used chemotherapeutic drugs was calculated to evaluate the NAC response. No significant differences were observed in the HER2-enriched subtype. **P < 0.01, *p < 0.05

To predict NAC treatment response outcomes in the study cohort, the half-maximal inhibitory concentration (IC50) was calculated. Sensitivity to multiple potential therapeutic drugs varied significantly among non-responders. Non-responders to Luminal A and LuminalB/HER2- showed greater sensitivity to SL.0101.1, whereas responders to LuminalB/HER2 + and HER2-enriched subtypes showed higher sensitivity (Fig. 6). SL.0101.1 is a selective ribosomal S6 kinase (RSK) inhibitor. LuminalB/HER2 + non-responders showed greater sensitivity to Nutlin-3a, whereas TNBC responders showed greater sensitivity. These therapeutic drugs could be suggested for NAC treatment.

## Discussion

RNA-seq analyses identified gene expression differences between NAC-resistant and NAC-sensitive patients, highlighting specific DEGs predictive of treatment resistance and overall survival. Numerous DEGs were found in non-responders, linked to carcinogenesis and tumor progression in BC and other models (Supplementary Table 2), but few were significant independent risk factors. As NAC response varies among breast cancer subtypes, early predictors are crucial for adjusting treatment strategies, reducing toxicity in responders, and enabling timely modifications in non-responders. Given the unique genetic and epidemiological characteristics of the Latin American population, particularly in Colombia, this study aimed to identify potential prognostic genes for predicting NAC nonresponse. Integrative transcriptome analysis has identified several genes, oncogenes, and pathways associated with non-responses, suggesting their involvement in tumorigenesis and their role in predicting therapeutic responses. Specifically, in the LuminalB/HER2- subtype, low expression of *APOD* and *GPR132*, along with overexpression of *CLEC3A*, were linked to non-response. In the LuminalB/HER2 + subtype, low *CSF3R* expression were potential independent predictors of non-response.

The apolipoprotein D (*APOD*) gene, a small circulating glycoprotein regulated by androgens and estrogens, is vital for cell proliferation, neovascularization, oxidative stress, inflammation, and the transport of small hydrophobic molecules (Zhao 2022; Zhao et al. 2023; Fyfe-Desmarais 2023). *APOD *binds strongly to arachidonic acid (ARA), a precursor of the signaling pathways that generate prostaglandins and leukotrienes, which are crucial for inflammatory responses and malignant transformation (Desmarais 2020. Elevated APOD expression is primarily observed in the glandular epithelium of the breast, with significant protein accumulation in the breast cysts (Sanchez 2021). While APOD can inhibit proliferation of BC cells and improve prognosis, its presence in stromal cells may promote cancer progression and worsen outcomes (Zhou^2020^; Li 2019). *APOD´s role * in gastric (Huo et al. 2022; Wang et al. 2024), thyroid (Ruchong et al. 2021), and cervical cancers (Jiang et al. 2021; Zhang et al. 2022) is associated with poor prognosis, although its mechanisms and therapeutic potential are not fully understood. Enrichment pathway analysis showed ARA enrichment in LuminalB/HER2- (Fig.5I) and risk score analysis indicated that a low-risk *APOD* model correlated with a better prognosis. Our predictive risk score model, validated by ROC and Kaplan-Meier curves (AUC > 0.6) using TCGA database data, suggests potential of *APOD* as a prognostic tool for NAC response in LuminalB/HER2- patients. The model demonstrated that the association of *APOD* with *GPR132* did not improve performance, highlighting the efficacy of *APOD* alone. Immunohistochemistry confirmed higher APOD expression in responders, aligned with the gene expression results, and indicated its potential as a predictive biomarker for LuminalB/HER2- patients. Commercial prognostic marker tests lack verification in Colombian populations. Our study investigated the overlap between these tests and DEGs using sequence analysis, revealing minimal overlap with Mammaprint and PAM50 panels, and no overlap with Oncotype Dx or EndoPredict, emphasizing the necessity for population-specific markers. *CDC42BPA* was significantly expressed in the luminal subtypes, particularly in the cell proliferation pathways.

Reduced *GPR132* expression is linked to a protective effect in LuminalB/HER2- patients, lowering the risk of poor prognosis. *GPR132* mediates tumor-macrophage interactions by detecting lactate in the acidic TME, promoting the M2-like macrophage phenotype. These interactions facilitate the adhesion, migration, invasion, and metastasis of cancer cells. Mouse studies have shown that *GPR132* deletion reduces M2 macrophages and limits lung metastasis of BC. Clinically, high *GPR132* levels correlate with increased M2 macrophages and poor outcomes in BC patients, highlighting the lactate-*GPR132*axis as a key metastasis driver and potential therapeutic target (Chen et al. 2017).

NAC is believed to facilitate metastasis by inducing cellular stress and remodeling the TME. Enrichment analysis revealed that Luminal A DEGs were mainly linked to the extracellular matrix (ECM) receptor, actin cytoskeleton regulation, and Pi3K-Akt signaling pathway. ECM molecules, receptors, and remodeling enzymes are vital for BC therapy resistance. ECM significantly aids tumor progression by fostering anoikis resistance and cell adhesion-mediated drug resistance (Wang et al. 2022). ECM’s role has been noted in other cancers (Wang et al. 2022). In glioblastoma, ECM within the brain’s TME promotes chemotherapy resistance, suggesting potential for novel matrix-targeted combination therapies (Xiao et al. 2020). Using Cytosig, we observed overexpression of IL-1β, IL-2, TGF-β1, and TGF-β2 in LuminalB/HER2 + non-responders. A plasma study indicated that effective NAC response correlated with increased IL-12 levels, which stimulates cytotoxic and pro-inflammatory mechanisms in CD8 + T cells (Osuna-gómez et al. 2021). This contradicts our findings based on *in silico* gene expression rather than cell analysis, warranting further studies. TGF-β acts as a tumor suppressor but can paradoxically promote invasiveness and metastasis via EMT (Katsuno et al. 2013). In BC, assessing TGF-β pathway protein levels may be useful for prognosis and identifying patients at higher recurrence risk (de Kruijf et al. 2013). Convolution models analyzing distinct immune subsets showed a low abundance of CD4 + T cells, indicating adaptive resistance via the TGF-β pathway. These findings underscore TME’s role in tumor development and growth by cytokine production from immune and non-immune cells, crucial for disease progression. TME evaluation is vital in clinical decisions, affecting chemotherapy efficacy and guiding precision-oriented BC care.

Studies have demonstrated that molecular pathways and immune responses significantly influence the efficacy of chemotherapy for BC. Enrichment analysis in LuminalB/HER2 + patients indicated the involvement of the PD-L1/PD-1 checkpoint and JAK-STAT signaling pathways, with the latter implicated in inducing PD-L1 expression in various cancers (Doi et al. 2017). Resistance to chemotherapeutic agents, such as paclitaxel, etoposide, and 5-fluorouracil, has been linked to pathways regulating PD-L1 expression, including JAK/STAT, MAPK, PI3K-AKT, and NF-κB in cancer cells, including human BC (Doi et al. 2017; Zhang et al. 2008). Tumor cells regulate PD-L1 expression through innate and adaptive immune resistance (Doi et al. 2017). Innate resistance involves the PI3K/AKT and STAT3 signaling pathways driving PD-L1 expression (Marzec et al. 2008), while adaptive resistance involves the JAK/STAT, PI3K, MAPK, and NF-κB pathways mediating PD-L1 upregulation in response to IFN-γ (SEO et al.^2005^). Convolution models analyzing immune subsets revealed low CD4 T cell and high macrophage M0 levels in non-responders, suggesting that PD-L1 upregulation mediates both resistance types. The Wnt/β-catenin and Notch signaling pathways are linked to BC development and regulation, highlighting the complex interactions between molecular pathways and the chemotherapy response (Gao et al. 2022; Zhou et al. 2020). Moreover, estrogen receptor alpha expression is associated with reduced sensitivity to chemotherapy in BC cells, indicating the role of hormone receptor pathways in treatment outcomes (Xie et al. 2020).

Recent studies have indicated a significant link between metabolism and oncogenesis, suggesting that metabolic changes may affect oncological treatment efficacy (Jiménez-Franco et al. 2024). In LuminalB/HER2- breast cancer, we observed alterations in glyoxylate and dicarboxylate metabolism and glycine, serine, and threonine metabolism. Preliminary metabolomic studies have shown beneficial metabolic changes in patients with BC post-surgery and adjuvant therapy, particularly in glyoxylate and dicarboxylate metabolism and glycine, serine, and threonine metabolism, especially post-radiotherapy (Jiménez-Franco et al. 2024). Comparative profiling of BC cells with different metastatic potentials identified pathways such as alanine, aspartate, and glutamate metabolism; glyoxylate and dicarboxylate metabolism; the pentose phosphate pathway; glycolysis or gluconeogenesis; and the tricarboxylic acid cycle that are associated with metastasis (Kim et al. 2016). Glyoxylate and dicarboxylate metabolism have also been linked to neoadjuvant chemotherapy resistance, as shown by studies using metabolomics and machine learning, highlighting its role in treatment resistance and metastasis (Cardoso et al. 2022; Kim et al. 2016).

Previous studies (Issa-Nummer et al. 2013; Ono et al. 2012; Solinas et al. 2017) have demonstrated that high stromal tumor-infiltrating lymphocytes (sTILs) correlate with a higher likelihood of achieving pCR during NAC in BC across molecular subtypes, with improved DFS in HER2-enriched and TNBC patients, but not in luminalB/HER2- cases. Cluster analysis of tumor-infiltrating immune cells (TIICs) identified two groups: C2, with pro-inflammatory cells (macrophages, B cells, T cells), high sTILs, better pathological responses, and no relapses, and C1, with low inflammatory infiltrates and poorer outcomes. C2 patients might be suitable for de-escalation therapy or immunotherapy, whereas C1 patients may benefit from NAC escalation to boost immune responses (Arqueros et al. 2024). In our study, responders showed a better immunoscore. These results suggest TIIC-based tumor classification could refine treatment strategies.

NAC for BC increases regulatory T cells and decreases CD8 + T cells within tumors, significantly altering the TME (Urueña et al. 2022). Our xCell analysis of HER2-enriched cases showed a notable decrease in CD8 + T cells in responders. CD8 + cytotoxic T lymphocytes are crucial predictors of response to anthracycline-based chemotherapy in breast cancer, underscoring the role of the immune system in treatment efficacy (Darwin et al. 2020; Deswangga and Alsoph 2018). We observed an increase in CD4 + T cells (naïve and memory) in the responders. Our findings and those of other studies confirm that CD4 + T cells likely include tumor antigen-specific subsets, contributing to tumor regression during chemotherapy (Péguillet et al. 2014), highlighting the intricate relationship between immune responses and therapeutic outcomes.

Our findings of elevated IL-4 levels in non-responders to neoadjuvant chemotherapy in LuminalB/HER2 + breast cancer align with previous research demonstrating IL-4’s role in promoting tumor survival and chemotherapy resistance. IL-4 has been shown to upregulate anti-apoptotic proteins like Bcl-xL and cFLIP, enabling tumor cells to evade chemotherapy-induced cell death (Todaro et al. 2008; Conticello^2004^). Moreover, IL-4 receptor overexpression supports tumor growth and resistance to treatment, further contributing to poor therapeutic response in these patients (Guruprasath et al. 2017). These findings position IL-4 as a key factor in chemotherapy resistance, underscoring its potential as a therapeutic target in this breast cancer subtype. Consequently, further studies are warranted to explore its clinical implications. The TME significantly impacts tumor development by producing cytokines from immune and non-immune cells, which are crucial for disease progression. Evaluating TME is crucial in clinical decisions, affecting chemotherapy effectiveness and guiding precision-oriented breast cancer care.

SL-0101 is the first specific inhibitor of p90 RSK, a regulator of processes, such as cancer cell proliferation and survival (Poomakkoth et al. 2016). Inhibition of RSK with SL-0101 may disrupt tumor-promoting signaling pathways, especially in lung cancer (Poomakkoth et al. 2016). Our results indicated a higher sensitivity to SL-0101 in non-responders to Luminal A and LuminalB/HER2 + subtypes. SL-0101 is a promising candidate for further cancer therapy research, potentially leading to new treatments that target RSK mechanisms and improve patient outcomes.

The limitations of this study include non-homogeneous samples among molecular subtypes and compromised RNA quality in FFPE samples due to oxidation, cross-linking, and chemical modifications from paraffin inclusion. Additionally, there is a lack of comprehensive data for accurately calculating residual cancer burden, and most HER2-positive patients did not receive the current standard of care—including targeted therapies such as trastuzumab—at the time of recruitment, limits our ability to fully assess molecular characteristics related to chemosensitivity in this subgroup. While bootstrap and jackknife resampling enhance the robustness of our findings, the small sample size (*n*= 19) in the Luminal B subtype limits generalizability. We performed 20 bootstrap iterations with two approaches to assess consistency, but caution is needed due to this small sample size. The jackknife method stabilizes results, although small sample sizes may introduce bias and limit precision (Zhang 2010; Taylor 1991). Despite assessing fold-change consistency through standard deviation, boxplots, and heatmaps, assay variability and outliers may still impact results. Robust clustering methods, such as jackknife distances, can identify co-regulated genes, but assay-specific biases must be considered in clinical applications. Furthermore, the lack of comprehensive genetic ancestry data linked to treatment response in publicly available cohorts restricted our ability to thoroughly assess the ethnic specificity and generalizability of the identified predictive biomarkers, thereby underscoring the importance of our data and research in addressing this critical aspect. Future research should include multicentric studies to validate, enhance, and expand upon our findings, thereby increasing their robustness and generalizability across diverse populations and molecular subtypes. While the study initially aimed to analyze all molecular subtypes, constraints related to sample size required a primary focus on the Luminal B subtype, thereby limiting the generalizability of the findings to other breast cancer subtypes. The predictive nature of our cytokine activity analysis, based on *in silico* data, presents limitations that should be acknowledged. Future studies should employ experimental validation using targeted methodologies and well-characterized samples to accurately elucidate the roles of cytokines, such as IL-4, in chemotherapy resistance. Nevertheless, the study’s strengths include a large sample size, a heterogeneous patient population, and the use of independent discovery and validation cohorts. Our findings support the increased use of NAC for BC in Colombia in recent years, providing valuable insights into treatment outcomes for this population.

These findings demonstrate that *APOD*, *GPR132*, *FGF10*, and *HBB* can serve as independent predictors of NAC response, and pathway analysis underscores the role of immune-related pathways in treatment outcomes. Validation of these results through immunohistochemistry and the observed drug sensitivities suggests that personalized treatment strategies could significantly improve patient outcomes. Further analyses should examine differential gene expression based on molecular subtype and genetic ancestry. Additionally, candidate gene expression requires validation in a larger cohort. If confirmed, these findings could elucidate mechanisms of therapy resistance and aid in selecting Latino (specifically Colombian) patients who would benefit from NAC.

## Conclusion

It is important to conduct studies with larger populations to validate these results and have potential in clinical practice. To validate its predictive function, analyses of other public databases were performed and similar results were obtained, confirming APOD´s role as a potential predictive biomarker. This study highlights the importance of identifying specific gene expression profiles and immune cell population changes to predict response to NAC in patients with breast cancer. This study offers preliminary data to identify genetic markers for stratifying patients at higher relapse risk requiring more intensive targeted interventions. Our findings may lead to more personalized and effective treatment strategies, ultimately improving the clinical outcomes and quality of life of patients in this region.

## Supplementary information

Supplementary Material 1.

## Data Availability

The datasets generated during the current study are available in the Gene Expression Omnibus (GEO) repository, [https://www.ncbi.nlm.nih.gov/geo/query/acc.cgi?acc=GSE280902]. The datasets supporting the conclusions of this article are available in the Gene Expression Omnibus (GEO) repository, [https://www.ncbi.nlm.nih.gov/geo/query/acc.cgi?acc=GSE20194], [https://www.ncbi.nlm.nih.gov/geo/query/acc.cgi?acc=GSE25066].

## Abbreviations

DEG: Differentially expressed gene
IC50: Half-maximal inhibitory concentration
NAC: Neoadjuvant chemotherapy
OS: Overall survival
pCR: Pathological complete response
TME: Tumor microenvironment
TNBC: Triple-negative breast cancer
