# Supplementary material for: Identification of predictive pretreatment biomarkers for neoadjuvant chemotherapy response in Latino invasive breast cancer patients
Source: Mol Med. 2025 Dec 9;31:335. doi: 10.1186/s10020-025-01338-8 (PMC12690809; doi:10.1186/s10020-025-01338-8)
Supplement: Supplementary file 1 — Supplementary Material 1. [file 10020_2025_1338_MOESM1_ESM.docx]

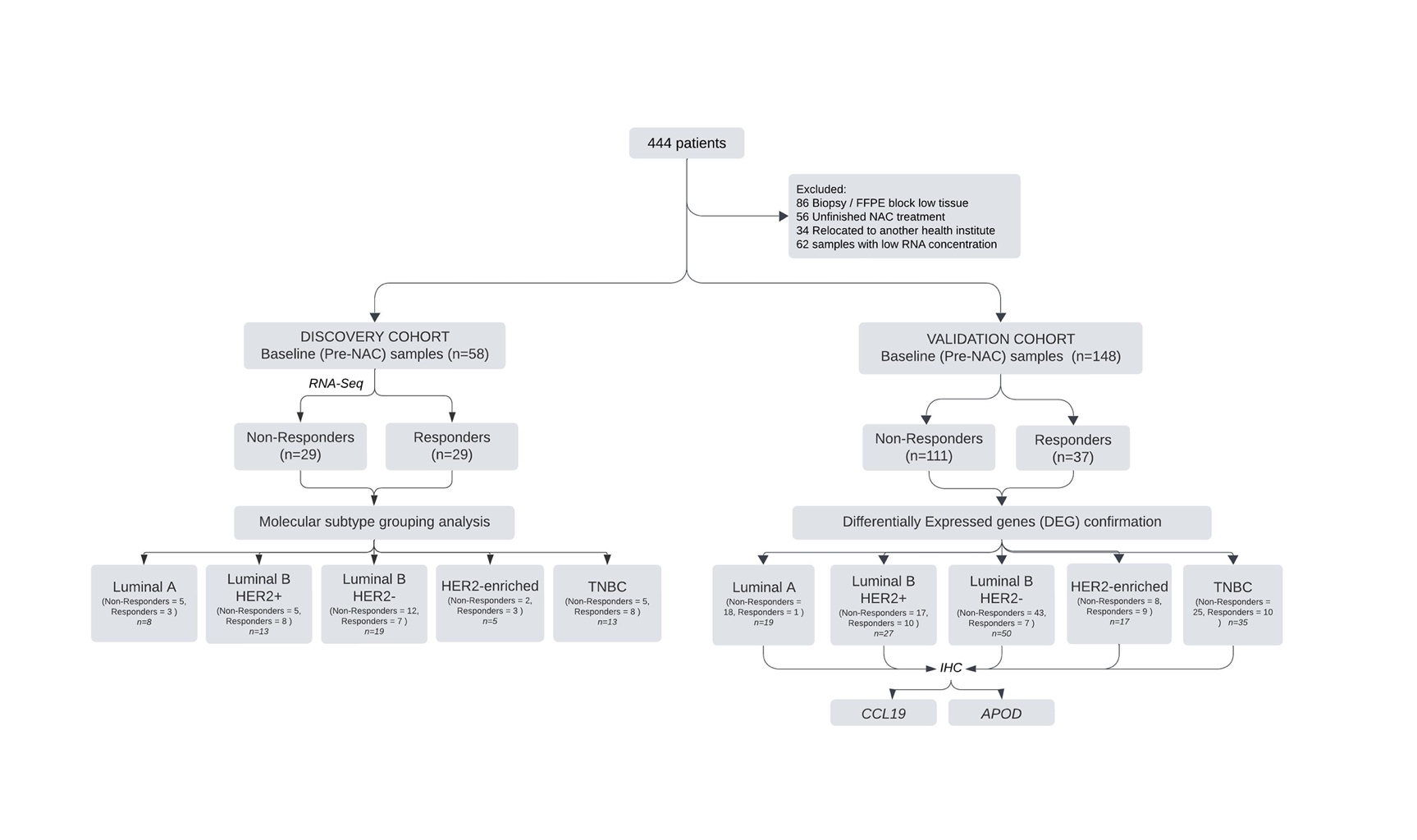


**Supplementary Figure 1.** Flow diagram of patient inclusion and molecular subtype groups. Patients were classified as responders if they exhibited pathological complete response (pCR) and as non-responders if they did not achieve pCR.

**Supplementary Table 1.** Demographic and clinicopathological characteristics of the patients with breast cancer Data are presented as number (percentage), unless otherwise indicated.

**Supplementary Table 2.** Differentially expressed genes (DEGs) by BC molecular subtype in non-responders compared with responders. Log2Fold Change (Log2FC).

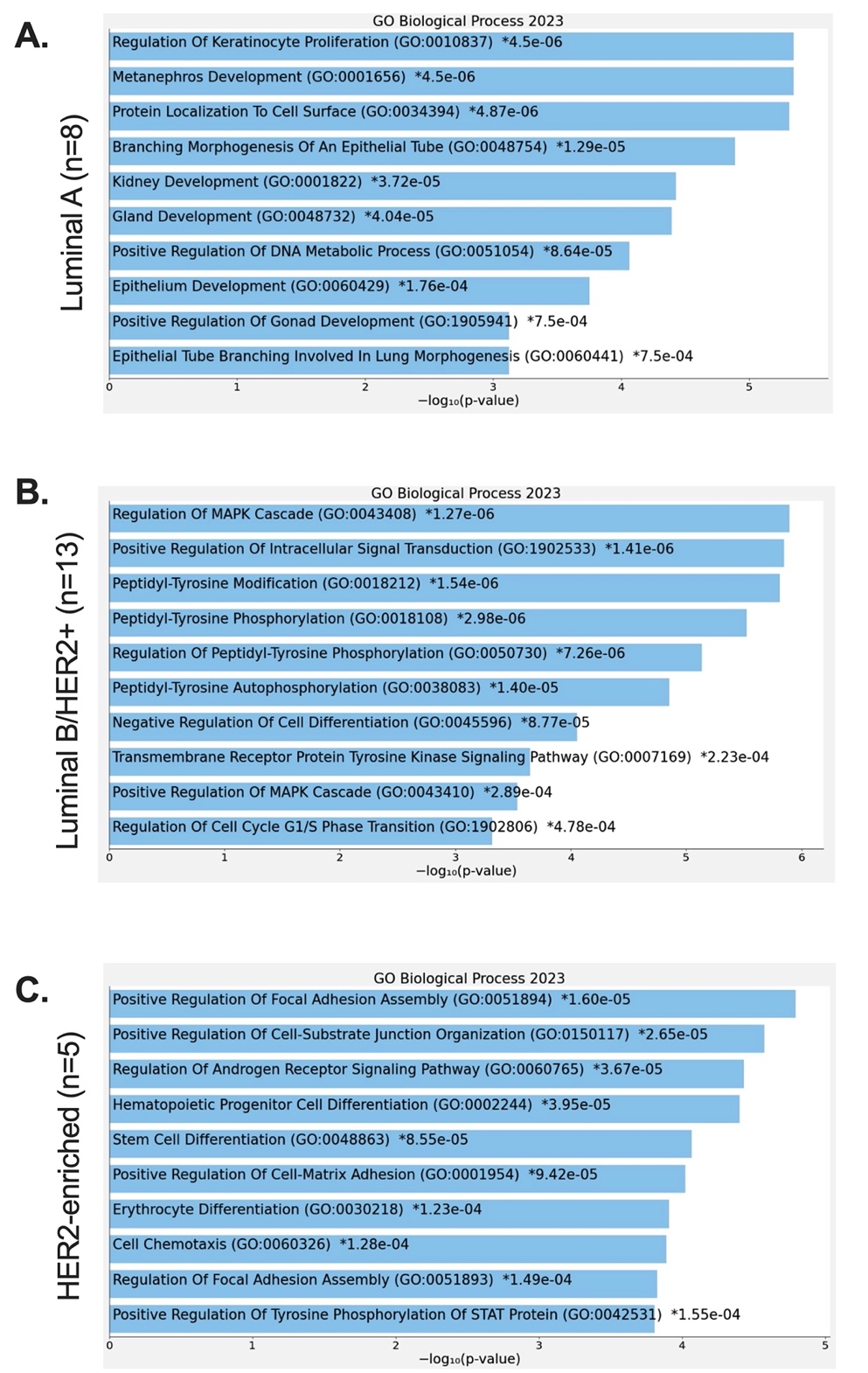


**Supplementary Figure 2.** Biological processes depicting common DEGs among molecular subtypes Luminal A (A), Luminal B/HER2+ (B) and Luminal B/HER2- (C). Biological process bar charts of the top enriched terms from the GO_Biological_Process_2023 gene set library. The top 10 enriched terms for the input gene set are displayed based on -log10(p-value), with the actual p-value shown next to each term. The term at the top had the most significant overlap with the input query gene set. LuminalB/HER2- and TNBC had low number of DEGs and no biological processes were observed.

**Supplementary Table 3.** Intersection of differentially expressed genes (DEGs) and gene signatures Mammaprint and PAM50.

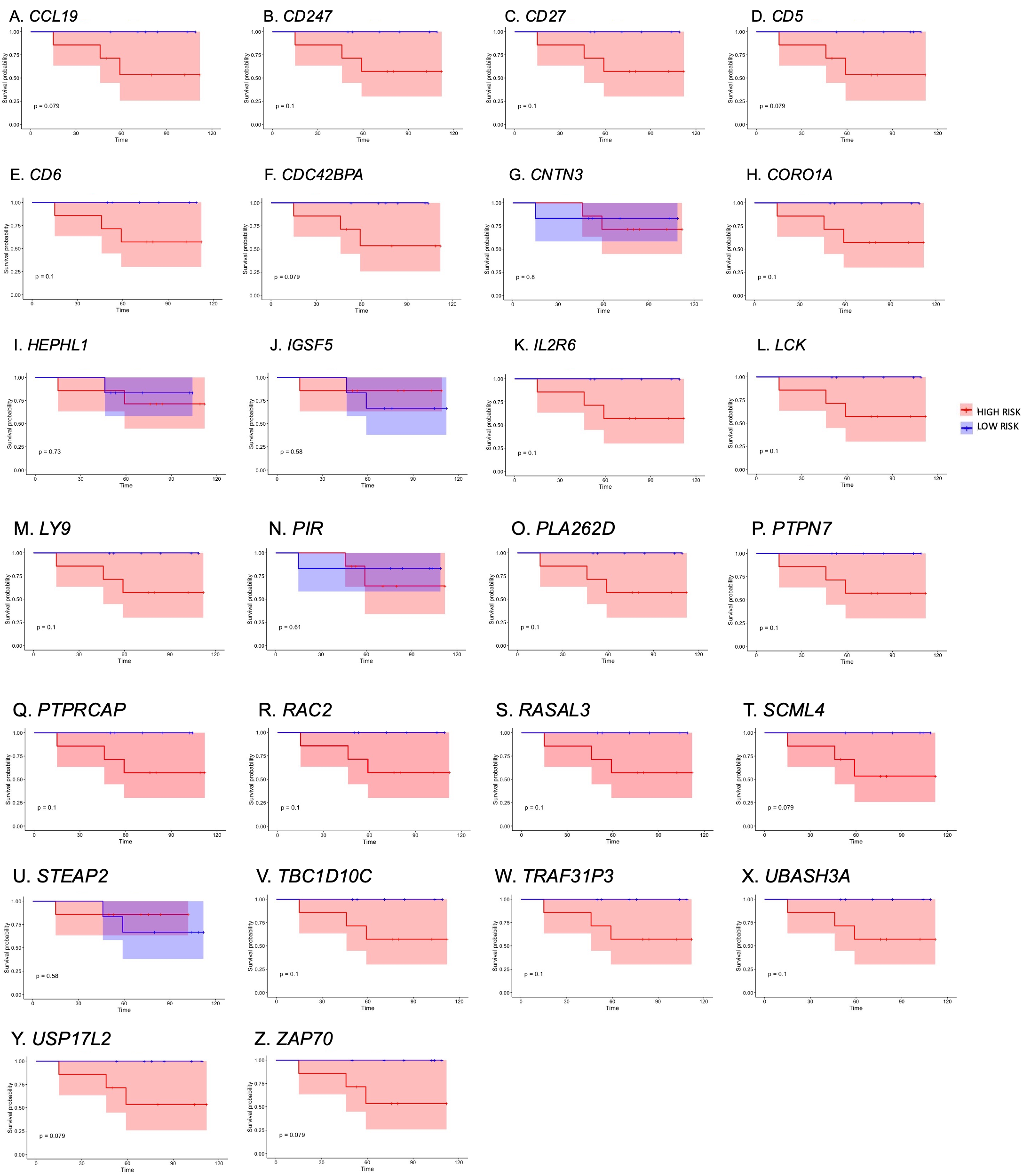


**Supplementary Figure 3.** Survival Analysis for LuminalB/HER2+ patients stratified into low- and high-risk groups based on the prognostic model. Although the differences were not statistically significant (*p* >0.05), there was a clear trend indicating that the high-risk groups were associated with poorer survival outcomes.


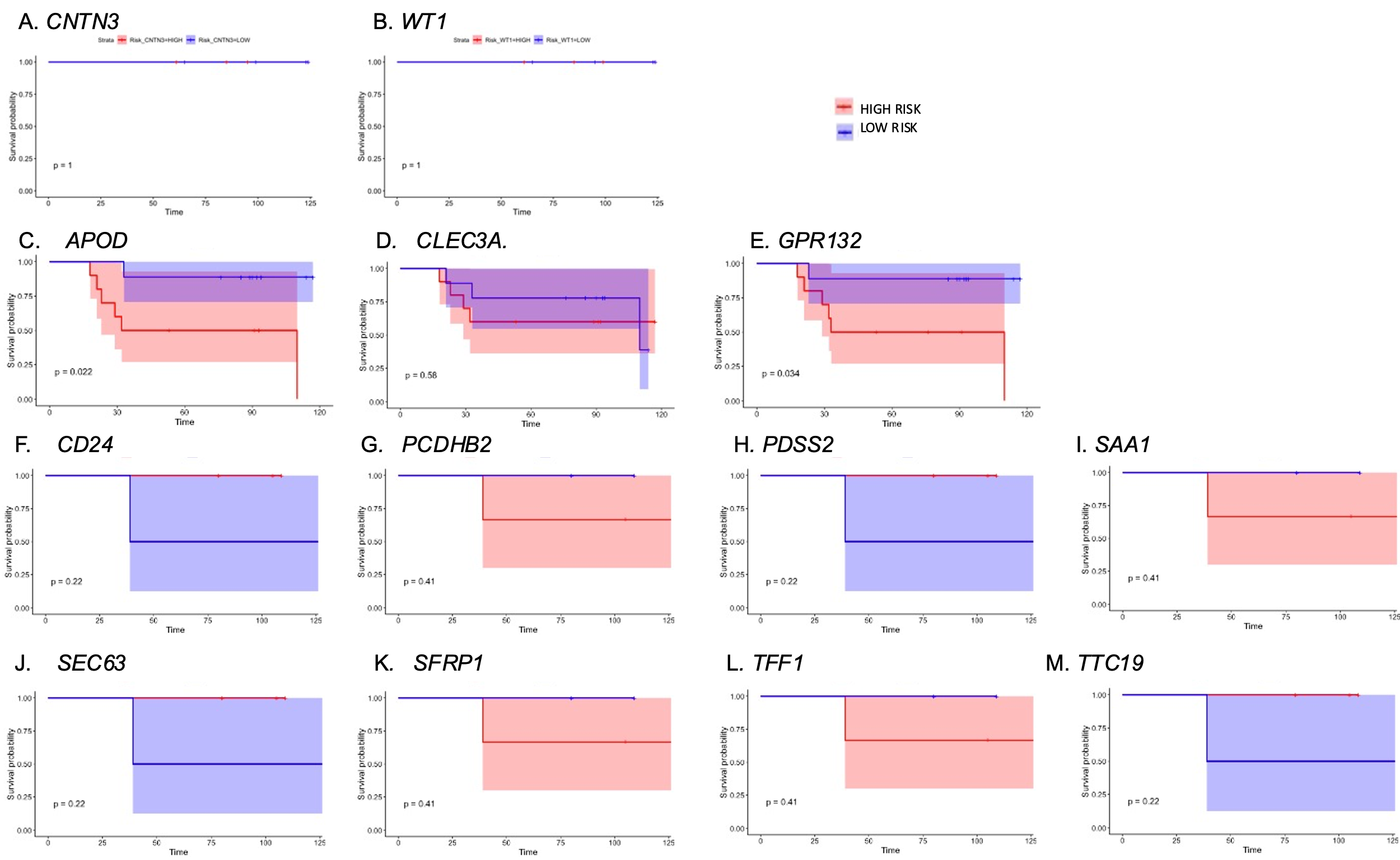


**Supplementary Figure 4.** Survival Analysis based on the prognostic model. (A, B) KM survival curves for Luminal A patients in the low- and high-risk groups for the *CNTN3* and *WT1* genes. (C-E) KM survival curve for LuminalB/HER2- patients for *APOD*, *CLEC3A,* and *GPR132* genes; (F-M) KM survival curve for HER2-enriched patients for *CD24*, *PCDHB2*, *PDSS2, SAA1, SEC63, SFRP1, TFF1,* and *TTC19* genes. The differences were statistically significant (*p* >0.05) for *APOD*, *CLEC3A* and *GPR132 expression*.

**Supplementary Table 4**. Univariate analysis to identify clinical variables related to gene expression in non-responders to NAC (A), including all and (B) each molecular breast cancer subtype. P-values for logistic regression variables were determined using the likelihood-ratio chi-squared test. Univariate logistic regression models incorporated clinical, demographic, and genetic features using the Wilcoxon rank-sum and Fisher's exact tests.


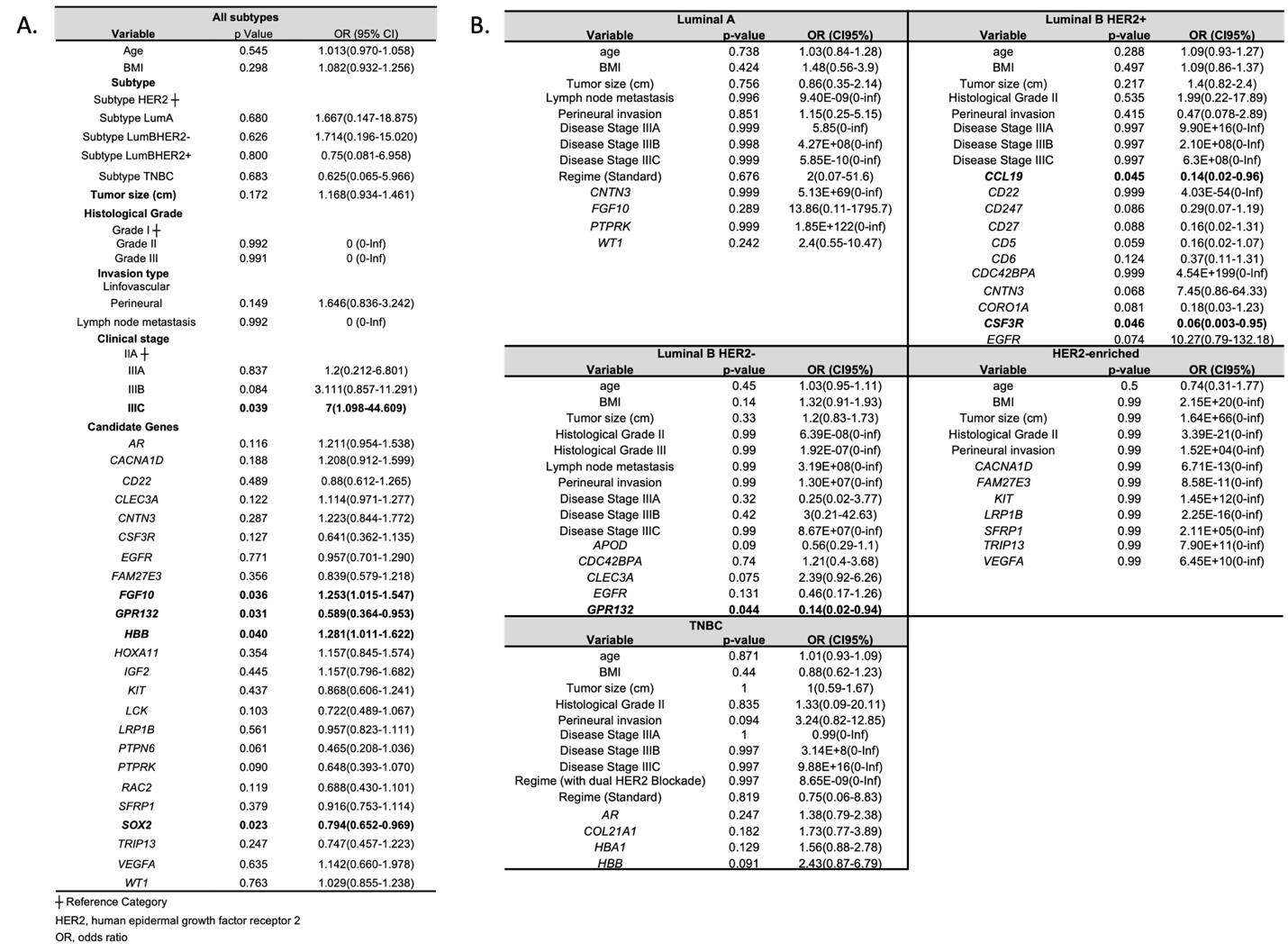


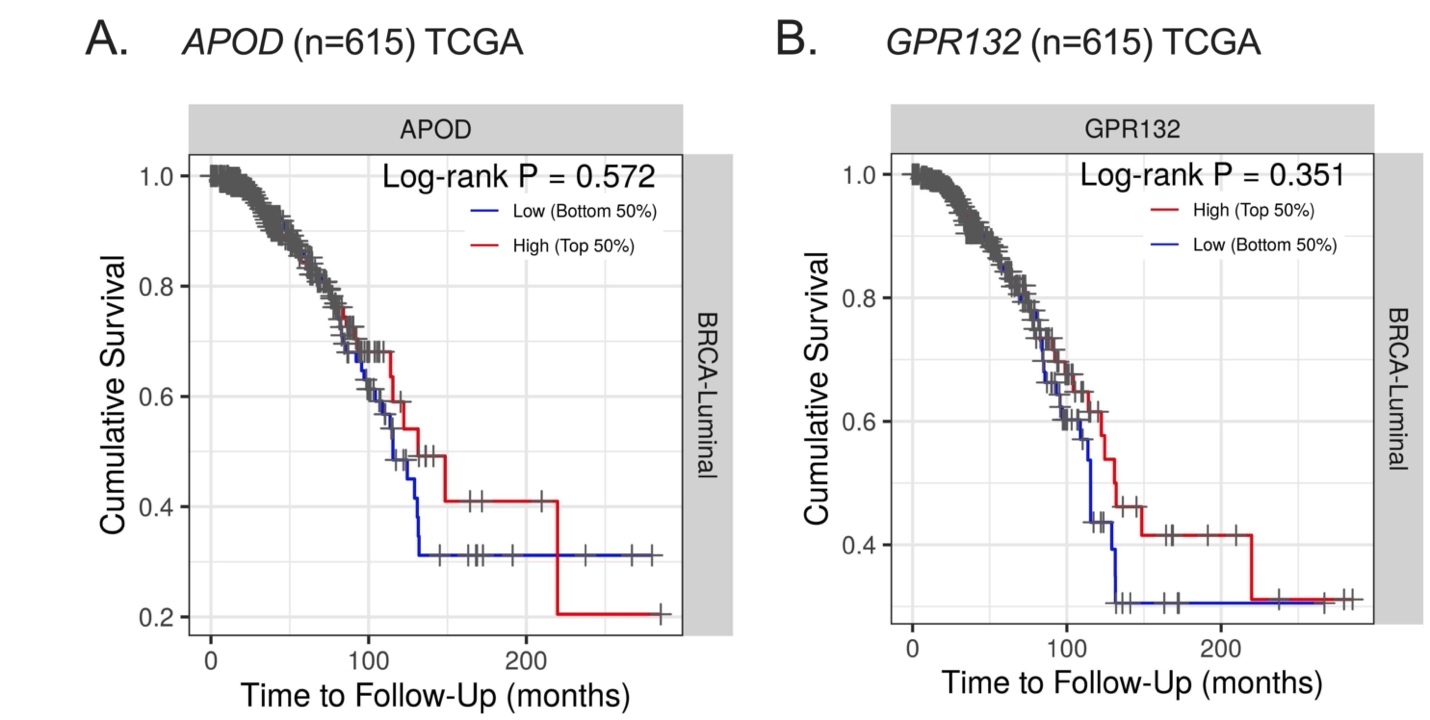


**Supplementary Figure 5.** Survival analysis based on the risk prognostic model. Kaplan-Meier survival curves for patients in the low- and high-expression groups within the Luminal TCGA BRCA cohort, categorized by the top and bottom 50% of gene expression levels. (A) *APOD*, (B) *GPR132*. Statistically significant differences were not observed (Log-rank P-values: *APOD* = 0.572, *GPR132* = 0.351).


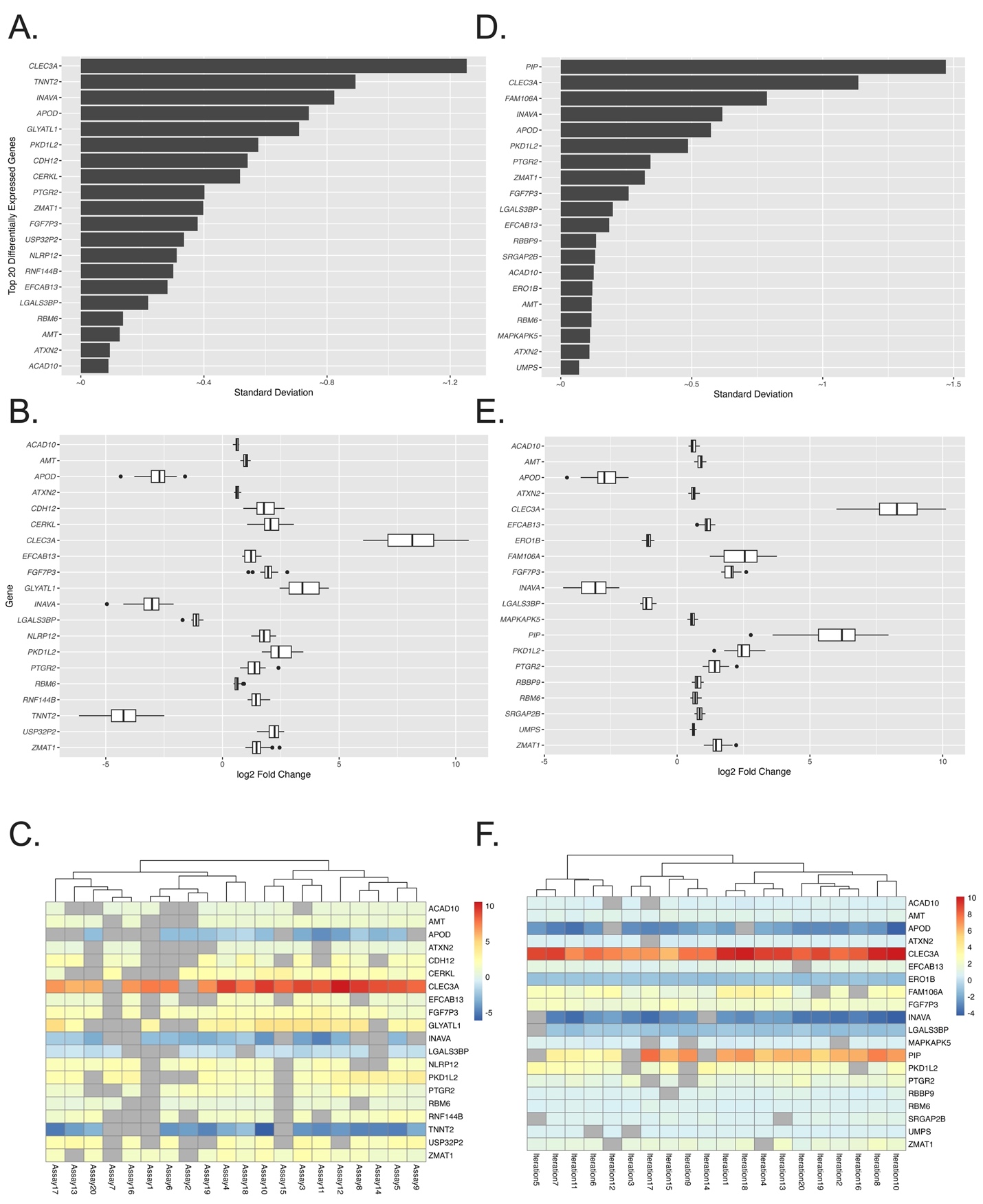


**Supplementary Figure 6**: Bootstrap Analysis of Gene Expression Variability (A, B, C) 6 non-responders and 6 responders (n=12); (D, E, F) 11 non-responders and 6 responders (n=17). (A, D) Bar plots of fold-change variability (standard deviation, SD) per gene, showing the consistency of fold-change values across 20 bootstrap iterations.

(B, E) Box plots of log2 fold changes per gene, illustrating the distribution of gene expression changes in non-responder and responder groups. (C, F) Heatmaps of gene expression data across assays, highlighting differential expression patterns across 20 bootstrap iterations for each gene. The intensity of colors represents the log2 fold change for each gene and assay.

**Supplementary Table 5.** Differential APOD expression in several datasets from the Gene Expression Omnibus (GEO) database.

******

**Supplementary Table 6.** Detailed functional analysis (significantly enriched pathways) of differentially expressed genes in baseline samples between non-responders and responders among different molecular subtypes.

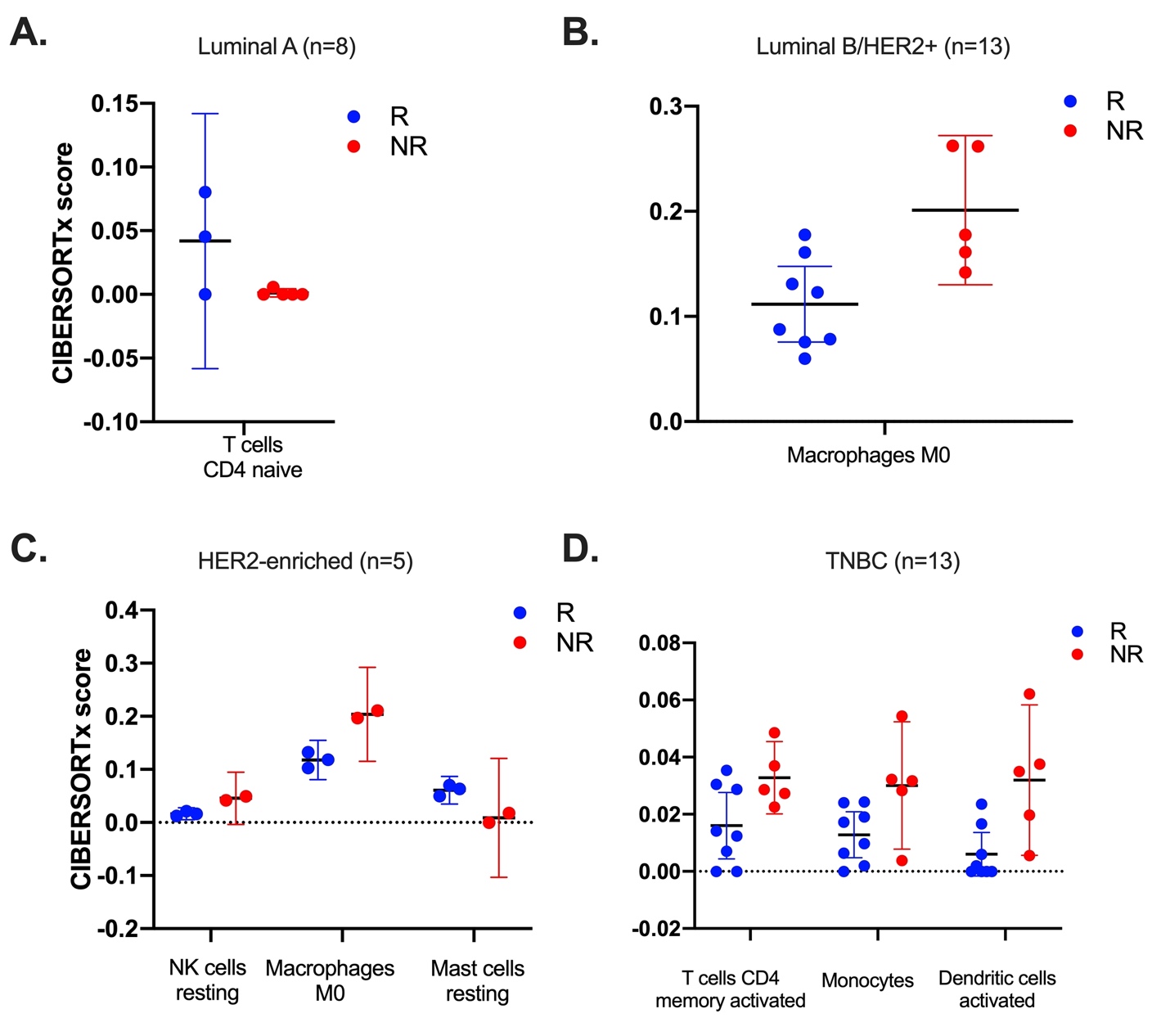


**Supplementary Figure 7.** Significant cellular heterogeneity landscape of tissue expression profiles among different molecular subgroups using CIBERSORTx approaches for Luminal A(A), LuminalB/HER2+ (B), LuminalB/HER2- (C), HER2-enriched (D) and TNBC (E). No significant differences were found between responders and non-responders for LuminalB/HER2-.

**Supplementary Table 7.** P-values from xCell cell types across comparison groups. Significant values (p<0.05) are shown in bold.
